# The relationship of hip fracture and thyroid disorders: a systematic review

**DOI:** 10.3389/fendo.2023.1230932

**Published:** 2023-10-10

**Authors:** SeyedAhmad SeyedAlinaghi, Soudabeh Yarmohammadi, Mohsen Dashti, Afsaneh Ghasemzadeh, Haleh Siami, Ayoob Molla, Sona Mahrokhi, Kowsar Qaderi, Ghazal Arjmand, Sahar Nooralioghli Parikhani, Masoomeh Fathi Amrollah, Peyman Mirghaderi, Esmaeil Mehraeen, Omid Dadras

**Affiliations:** ^1^ Iranian Research Center for HIV/AIDS, Iranian Institute for Reduction of High-Risk Behaviors, Tehran University of Medical Sciences, Tehran, Iran; ^2^ Trauma Research Center, Kashan University of Medical Sciences, Kashan, Iran; ^3^ Department of Radiology, Tabriz University of Medical Sciences, Tabriz, Iran; ^4^ School of Medicine, Islamic Azad University of Medical Sciences, Tehran, Iran; ^5^ School of Medicine, Bushehr University of Medical Sciences, Bushehr, Iran; ^6^ Department of Midwifery, School of Nursing and Midwifery, Kermanshah University of Medical Sciences, Kermanshah, Iran; ^7^ School of Medicine, Shahid Beheshti University of Medical Sciences, Tehran, Iran; ^8^ School of Medicine, Tehran University of Medical Sciences, Tehran, Iran; ^9^ Students’ Scientific Research Center (SSRC), Tehran University of Medical Sciences, Tehran, Iran; ^10^ Department of Health Information Technology, Khalkhal University of Medical Sciences, Khalkhal, Iran; ^11^ Bergen Addiction Research, Department of Addiction Medicine, Haukland University Hospital, Bergen, Norway

**Keywords:** hip fracture, thyroid disease, thyroid disorder, thyroid dysfunction, thyroid

## Abstract

**Introduction:**

Bone density regulation is considered one of the systems affected by thyroid hormones, leading to low bone density that can result in pathologic fractures, including hip fractures. This review aimed to update clinicians and researchers about the current data regarding the relationship between hip fractures and thyroid disorders.

**Methods:**

English papers were thoroughly searched in four main online databases of Scopus, Web of Science, PubMed, and Embase. Data extraction was done following two steps of screening/selection using distinct inclusion/exclusion criteria. This study used the Preferred Reporting Items for Systematic Reviews and Meta-Analysis (PRISMA) checklist and the Newcastle-Ottawa Scale (NOS) as bias assessment.

**Results:**

In total, 19 articles were included in the research. The risk of hip fractures in women with differentiated thyroid cancer (DTC) is higher than hip fractures caused by osteoporosis. Men with hyperthyroidism and subclinical hyperthyroidism are at higher risk for hip fracture. Also, a decrease in serum thyroid stimulating hormone (TSH) may be associated with an increased risk of hip fracture.

**Conclusion:**

Reaching a consensus conclusion regarding the association between subclinical thyroid dysfunction and hip fracture is not feasible due to the heterogenicity of evidence; however, there may be a higher risk of fracture in individuals with subclinical hyperthyroidism.

## Introduction

Regulating metabolism and cell adjustment are just examples of what thyroid hormones do in the human body. Changes in these hormone levels occur in hypothyroidism, hyperthyroidism, subclinical hypothyroidism, and subclinical hyperthyroidism (1). Hypothyroidism is a common endocrine disorder caused by autoimmune thyroiditis (Hashimoto thyroiditis), iodine deficiency, or following surgery or radioiodine therapy (2). Hyperthyroidism is defined by elevated circulating free thyroid hormones, and overt hyperthyroidism is recognized as a low bone density or osteoporosis risk factor in older women. However, the relationship between biochemically defined subclinical hypothyroidism or hyperthyroidism and fracture risk is unknown. Still, in patients with subclinical hyperthyroidism, studies have shown that minor changes in thyroid hormone and/or thyroid stimulating hormone (TSH) levels can worsen bone mineral density (BMD) (3).

The bone remodeling cycle is what we call a continuous process of bone formation and bone resorption throughout the lifetime, and apart from local factors from osteoblasts and osteoclasts, the bone remodeling process is regulated by systemic factors such as calcitonin, parathyroid hormone (PTH), vitamin D3, estrogen, thyroid hormones, glucocorticoids, and growth hormones (4). T3 hormone increases bone formation through TRα receptors on osteoblasts and osteoclasts, but it can also increase osteoclast formation and the resorption process (5). Additionally, TSH action on the TSHR found in both osteoblasts and osteoclasts can also affect the bone remodeling cycle like T3 (6).

Changes in these hormone levels greatly affect bone metabolism and density and can lead to a decreased bone mineral density (BMD) that presents as osteoporosis. About 30–40% of osteoporosis patients are at great risk of osteoporotic bone fractures with a high mortality risk. The most frequent osteoporotic fractures are vertebral, distal radius, and hip fractures. Vertebral and hip fractures are considered life-threatening pathologies in the elderly (3). Hip fractures are a significant and incapacitating condition that disproportionately affects older women (7–15). While the epidemiology of hip fractures varies across countries, it is estimated that approximately 18% of women and 6% of men globally will be affected by this condition. Although the age-standardized incidence rate has decreased in many nations, the aging population generates a much greater impact (7–15). Therefore, the number of hip fractures globally is expected to swell from 1.26 million in 1990 to 4.5 million by the year 2050. The financial burden associated with this ailment is colossal since it requires long hospital stays and subsequent rehabilitation. Additionally, hip fracture is correlated with other adverse effects such as disability, depression, and cardiovascular diseases, which further exacerbates societal costs (7–15).

This review aimed to update clinicians and researchers about the current evidence regarding the relationship between hip fractures and thyroid disorders.

## Methods

According to the Preferred Reporting Items for Systematic Reviews and Meta-Analysis (PRISMA), this systematic review was carried out (16). The Newcastle-Ottawa Scale (NOS) quality assessment tool was used to evaluate methodological quality.

### Data sources

Systematic searches were conducted in Embase, PubMed, Scopus, and Web of Science databases without time limitation. Manual checks were made for any additional studies bibliography of relevant studies.

**The following keywords were used in combination:**

A: “Hip fracture” OR “Trochanteric fracture” OR “Intertrochanteric fracture” OR “Sub trochanteric fracture” OR “Femoral fracture” [Title/Abstract]
B: “Thyroid disease” OR “Thyroid disorder” OR “Thyroid dysfunction” [Title/Abstract]
C: [A] AND [B]

### Study selection

In two stages of screening and selection, publications of interest were included. First, titles and abstracts were evaluated, and relevant publications were chosen for the second stage. This step involved reading through the complete text of these papers. Studies were selected for analysis using the following inclusion and exclusion criteria:

1. Studies that addressed hip fractures and thyroid disorders.
2. Original articles.
3. English studies.

**Exclusion criteria:**

1. A systematic review, meta-analysis, qualitative studies, case report, and letter to the editor.
2. Articles that do not have full text, or in a language other than English.

### Data extraction

For data extraction, the records were divided among four impartial assessors to retrieve the following details: study type, nation, first author, publication year, target population, comparison, and data on bone metabolism, including biochemical parameters, parameters of bone damage, and fracture data.

### Quality assessment and risk evaluation

The study’s methodological quality was assessed using the NOS. It focused on three areas, including participant selection (0-4 points), comparability of study groups (0-2 points), and ascertainment of exposure (0-3 points), containing eight questions with a total score of nine. Finally, based on the total number of stars received, each study was assigned one of three grades: excellent, fair, or poor. When a study received 3 or 4 stars in the selection domain, 1 or 2 stars in the comparability domain, and 2 or 3 stars in the outcome/exposure domain, it was considered to have “excellent” quality. In the selection domain, “fair” was used for 2 stars, in the comparability domain for 1 or 2 stars, and in the outcome/exposure domain for 2 or 3 stars. “Poor” was used when the selection domain, comparability domain, or outcome/exposure domain received 0 stars, 1 star, or no stars, respectively (**Table 1**). Also, this review study complies with the PRISMA checklist to increase soundness and reliability (35).

**Table 1:** Newcastle-Ottawa Scale (NOS) bias risk assessment of the study.

The first author (reference)	Selection (out of 4)	Comparability (out of 2)	Exposure/Outcome (out of 3)	Total (Out of 9)
Polovina et al. (17)	2	2	2	6
Vera et al. (18)	2	2	2	6
Lee et al. (19)	2	2	2	6
Bauer et al. (20)	3	2	2	7
Cauley et al. (21)	3	2	3	8
Gallagher et al. (22)	2	1	2	5
Polovina et al. (23)	2	2	2	6
Abrahamsen et al. (24)	3	2	2	7
Nguyen et al. (25)	3	1	2	6
Ahmad et al. (26)	2	2	2	6
Siru et al. (27)	3	2	3	8
Solomon et al. (28)	2	2	2	6
Svare et al. (29)	4	2	3	9
Waring et al. (30)	4	2	2	8
G. P. Leese (31)	4	1	2	7
Jennifer S. Lee (3)	4	2	2	8
Bo Abrahamsen (32)	4	2	2	8
L.J Melton III (33)	3	1	3	7
Margaret C. Garin (34)	4	2	3	9

## Results

**Figure 1:**
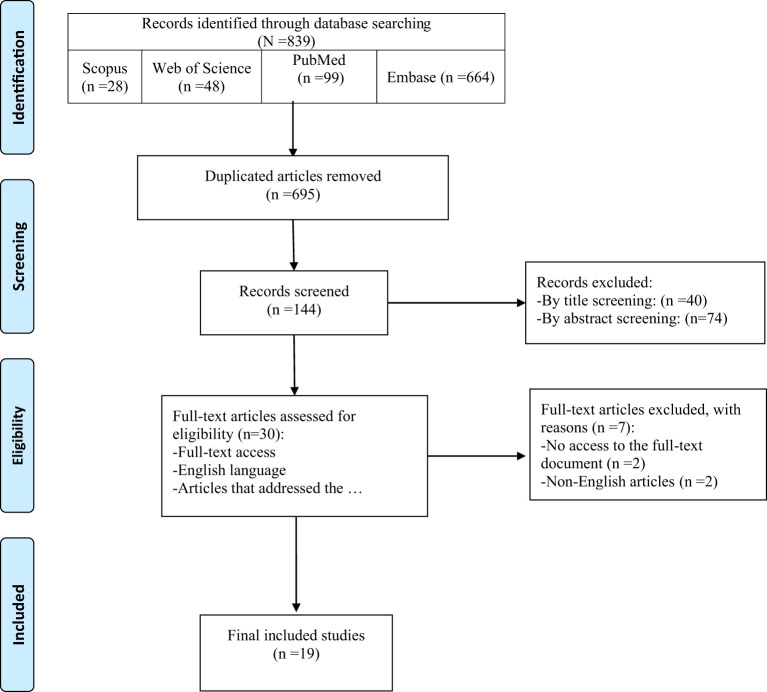
PRISMA 2020 flow diagram of the study retrieval process.

Among 839 records identified by the search, nineteen studies were included in this review (**Figure 1**). **Table 2** provides an overview of the included studies and the extracted data. A total of 15 cohorts and 4 cross-sectional studies reported the data of 229,294 males and 2,838,789 females.

**Table 2:** Description of the findings reported in eligible studies.

ID	The first author (reference)	Country	Study type	Study population (n=) Female (), Male()	Age Mean ± SD	Type of thyroid disorder	Thyroid disorder symptoms	Sites of fracture	Hip fracture rate Mean ± SD/Percent	Hip fracture symptoms	Relationship between thyroid disorders and hip fracture in Female/Male (Yes or No)	History of thyroid disorder	Relationship between thyroid disorder and hip fracture Adj HR/CI	Other risk factors for fracture	Drug used
1	Polovina et al. (17)	Serbia	Cross-sectional	Case: Female (27) Control: Female (51)	58.85 ± 7.83	autoimmune thyroid disease or toxic goiter	NR	Vertebral and hip fracture FRAX score	Hip fracture risk in the group with subclinical hyperthyroidism was 1.33 ± 3.92 *vs* controls 0.50 ± 0.46 (*p* = 0.022).	NR	Yes	None	-0.208 (-0.413, 0.004)	previous fractures, smoking status, alcohol consumption, parental fractures, MBI, fat mass, diabetes mellitus, and the onset of menopause	no steroid therapy longer than 6 months
2	Vera et al. (18)	Italy	Cohort	Case: Female (74) Control: Female (120)	51.9 ± 12.0	differentiated thyroid cancer (DTC)	NR	hip fracture and major osteoporotic fracture (MOF)	FRAX hip fracture: Baseline; 1.2 ± 2.0/0.6, Second evaluation; 1.9 ± 3.2/1.1 FRAX hip fracture in fracture pts: baseline; 3.5 ± 3.8/1.9, second evaluation; 4.6 ± 3.9/2.9	NR	Yes In DTC women, significant changes in FRAX were found, with a higher increase in the probability of hip fracture than of MOF.	NR	NR	Menopausal status, BMI, smoking status, Disease-free for DTC recurrence, diseases involving bone, Calcium/vitamin D supplementation, Anti-resorptive therapy	levothyroxine
3	Lee et al. (19)	Korea	Cross-sectional	Female (674) Male (343)	71.6 ± 4.7	euthyroidism	NR	hip fracture, vertebral fracture, and non-vertebral fracture	Female (4.5 ± 3.6) Male (2.1 ± 1.7)	NR	Female (Yes) Male (No) Lower TSH levels in the euthyroid range are related to lower bone mineral density BMD and weaker femoral structure in elderly women.	NR	NR	Menopausal status, BMI, smoking status, Drinking status, and hormone replacement	NR
4	Bauer et al. (20)	USA	Cohort	Female (1209)	Hip: Fracture (75.3 ± 6 5.6), No-fracture (71.7 ± 6 5.3) Vertebral: Fracture (73.2 ± 6 5.6), No-fracture (71.3 ± 6 5.0) Any non-spine: Fracture (72.8 ± 6 5.4), No-fracture (71.6 ± 6 5.2)	Hyperthyroidism	NR	hip fracture, vertebral fracture, and any non-spine fracture	2.0 ± 6 2.5	NR	Yes Women older than 65 with low serum TSH levels, indicating physiologic hyperthyroidism, are at increased risk for new hip and vertebral fractures. Use of thyroid hormone itself does not increase the risk for fracture if TSH levels are normal.	Previous hyperthyroidism or Graves disease,	relative hazard 3.6 (1.0–12.9)	Weight, history of hyperthyroidism, use of thyroid hormones, and use of oral estrogen	NR
5	Cauley et al. (21)	USA	Cohort	5994 Males No hip fracture (5698) Hip fracture (178)	Hip fracture (77.81 ± 6.08) No hip fracture (73.48 ± 5.81)	Hyperthyroidism	NR	Hip fracture	7 (3.93)	NR	Yes	NR	2.86 (1.32, 6.20)	demographic, lifestyle (alcohol consumption (average number of drinks per week), smoking, and dietary intake), personal and family medical history, functional status, anthropometric, cognitive, visual, and neuromuscular function	NR
6	Gallagher et al. (22)	USA	Cohort	Male (2) Female (11)	Median (78)	Thyrotoxicosis	NR	Hip fracture	NR	NR	Yes	NR	Male: 5.0 (0.6- 18.0) Female: 2.1 (1.04-3.7) Total: 2.3 (1.2-3.9)	cortisone therapy, radiotherapy to the pelvis, diabetes, rheumatoid arthritis, hemiplegia, hyperthyroidism, malabsorption syndrome, and gastric surgery	NR
7	Polovina et al. (23)	Serbia	Cross-sectional	Female (189)	Euthyroid: TPOAb- (60.46 ± 6.53), TPOAb+ (61.13 ± 7.10) Subclinical hyperthyroid: TPOAb- (59.63 ± 6.42), TPOAb+ (58.41 ± 7.72)	Autoimmune thyroid disease	NR	hip fracture and major osteoporotic fracture	TPOAb-: 1.06 ± 2.11 TPOAb+: 1.00 ± 1.18	NR	Yes Lower bone mineral density and FRAX scores for hip and osteoporotic fractures were associated with the presence of TPOAb in euthyroid postmenopausal women	None	T-score: 0.350 (0.189-0.651) FRAX: 2.053 (1.336-4.325)	BMI, fat mass, menopausal status, smoking status, diabetes mellitus, parental fractures, previous fractures, vitamin D level TSH was a better predictive factor for fractures in women with subclinical hypothyroidism	NR
8	Abrahamsen et al. (24)	Denmark	Cohort	Elevated TSH: Male (2386), Female (6027) Normal TSH: Male (99738), Female (122400)	Elevated TSH (54.3) Normal TSH: 50.2	Hypothyroidism	NR	hip fracture and major osteoporotic fracture	Female: 18-49, 0.21 (0.06–0.53); 50-74, 3.6 (2.8–4.5) Male: 18-49, 0.5 (0.1–1.3); 50-74, 2.9 (1.7–4.5)	NR	Yes	None	Baseline TSH value >4 mIU/L: All, 0.90 (0.80–1.02); Female, 0.94 (0.82–1.08); Male, 0.70 (0.51–0.97) Thyroxine prescription: All, 0.93(0.76–1.15); Female, 0.99 (0.79–1.24); Male, 0.60 (0.33–1.11) subsequent 6-month periods with low TSH >4 mIU/L: All, 0.99 (0.95–1.03); Female, 0.99 (0.95-1.03); Male, 0.96 (0.87–1.07) subsequent 6-month periods with low TSH< 0.3 mIU/L: All, 1.09 (1.04–1.15); Female, 1.10 (1.03–1.16); Male, 1.08 (0.93–1.25)	Previous fracture, history of comorbid conditions, and using medication such as Prednisolone or Osteoporosis medications	Thyroxine and subsequent 6-month periods with low TSH
9	Nguyen et al. (25)	USA	Cohort	Male (136)	Median age (43)	thyroidectomy	NR	thoracic or lumbar vertebra, proximal humerus, distal forearm, pelvis, or proximal femur fracture	NR	NR	Yes	Thyroid adenoma, goiter, and hyperthyroidism	the relative risk of any fractures for thyroidectomies patients Versus their controls was increased 1.5-fold (95% CI, 0.7–3.2).	Age at thyroidectomy, Extent of surgery, Extent of surgery, hyper/hypothyroidism, thyroid replacement, smoking status, ethanol use, and obesity	NR
10	Ahmad et al. (26)	Pakistan	Cohort	Hypothyroid: Female (27), male (8) Euthyroid: Female (395), Male (917)	Median ± IQR Hypothyroid (60 ± 29) Euthyroid (42 ± 32)	hypothyroidism	NR	Proximal Femur, Proximal Humerus, and Distal Radius and/or Elbow	29%	NR	Low-energy trauma more likely occurred in hypothyroid (71%) compared to 32% of euthyroid subjects (*P* < 0.001).	NR	NR	NR	NR
11	Siru et al. (27)	Australia	Cohort	Euthyroid: male (3117) Subclinical hypothyroidism: male (135) Subclinical hyperthyroidism: Male (86)	Euthyroid: 76.71 ± 3.47 Subclinical hypothyroidism: 77.78 ± 3.89 Subclinical hyperthyroidism: 77.27 ± 4.01	subclinical hyper - and hypothyroidism	NR	Hip fracture	NR	NR	No In euthyroid older men, TSH and FT4 were not associated with BTMs or incident hip fracture.	NR	Subclinical hypothyroidism: 1.50 (0.73 -3.07) Subclinical hyperthyroidism: 1.62 (0.71 -3.69)	BMI, WHR, smoking status, alcohol use, vigorous activity, hypertension, dyslipidemia, diabetes, CVD, cancer, frailty, creatinine status, and vitamin D status	
12	Solomon et al. (28)	USA	Cross-sectional	Female (300)	73 ± 12	Goiter, thyroid cancer, hypothyroidism, hyperthyroidism, thyroid nodules	NR	Hip fracture, spine fracture, forearm fracture	10.8%	NR	Yes	Women with a history of Hyperthyroidism and thyroid cancer had their first fracture earlier (p < 0.01) than women without thyroid disease.	there were no significant differences between women with thyroid disease and women without thyroid disease groups in the number or type of fractures.	Weight and height, smoking status, Menstrual/obstetrical status	Thyroxine women taking thyroid hormone for a variety of thyroid disorders do not appear to have an enhanced prevalence of hip, vertebral, or forearm fractures, but women with a history of hyperthyroidism may have a the propensity for their fractures to occur earlier in life
13	Svare et al. (29)	Norway	Cohort	Female (16610) Male (8595)	NR	Hyperthyroidism and Hypothyroidism	NR	ulnar and radial forearm fractures and hip fracture	NR	NR	No statistically significant relation between baseline TSH and subsequent fracture risk, but the data suggest a weak positive association with hip fracture risk among women with both low and high TSH	None	Female: TSH <0.5 (1.30 (0.87–1.94)), TSH>3.5 (1.19 (0.93–1.52)), TSH >4.0 and TPOAb-negative (1.87 (1.11–3.16)), TSH >4.0 and TPOAb-positive (1.75 (1.24–2.46)) Male: TSH <0.5 (0.99 (0.40–2.43)), TSH>3.5 0.64 (0.37–1.09	BMI, smoking status, and Recreational physical activity	NR
14	Waring et al. (30)	USA	Cohort	Male (1817)	Nonspine fracture: Yes (75.4 ± 6.4), No (73.6 ± 5.9) Hip fracture: yes (78.1 ± 6.1), No (73.6± 5.8)	Subclinical hyper/hypothyroidism	NR	Nonspine fracture and Hip fracture	Subclinical hyperthyroid: 1 ± 4.8 Subclinical hypothyroid: 4 ± 6.5	NR	There was no association between TSH or FT4 and bone loss, and fracture risk did not Differ r significantly by thyroid function category	high thyroid or Graves’ disease or low thyroid	Subclinical hyperthyroid: 0.63 (0.15–2.69) Subclinical hypothyroid: 0.75 (0.40–1.41) although neither TSH nor FT4 is associated with bone loss, lower serum TSH may be associated with an increased risk of hip fractures in older men	BMI, health status, physical activity status, smoking status, alcohol consumption, Oral corticosteroid use Participants who experienced hip fractures had a significantly lower BMI (p<0.001), lower physical activity score (p=0.01), were more likely to report a history of “high thyroid” or “Graves’ disease” (p=0.05), and consumed, on average, more alcoholic drinks per week (p<0.001) than those without hip fractures.	NR
15	G. P. Leese (31)	Scotland	cohort	female (1062) male (118)	NR	hypothyroid	NR	Hip/neck of femur	NR	NR	No There was no increase in risk for overall fracture, or fractured neck of femur in those on thyroxine with suppressed or normal TSH.	NR	There was no excess of fractures in patients on L-thyroxine even if the TSH is suppressed.	NR	L-thyroxine
16	Jennifer S. Lee (3)	USA	cohort	female (2270) male (1408)	72.8 ± 5.6	Subclinical hyperthyroidism or hypothyroidism	NR	NR	NR	NR	YES for men NO for women Older men with subclinical hyperthyroidism or hypothyroidism are at increased risk for hip fracture. Whether treatment of the subclinical syndrome reduces this risk is unknown.	NR	Men with subclinical hypothyroidism had a multivariable-adjusted HR of 2.31 (95% CI, 1.25-4.27); those with subclinical hyperthyroidism, 3.27 (0.99-11.30)./There was no association between subclinical thyroid dysfunction and hip fracture in women.	Thyroid function/BMI/ Age/Sex/Alcohol use/Cigarette smoking/Thiazide use/Diabetes mellitus/Age at menopause/Estrogen use/Calcium supplement intake/Physical activity/Frailty status/Antithyroid or corticosteroid medication/Thyroid hormone medication/Antiosteoporosis medication	Thyroid hormone medication/ Antithyroid or corticosteroid medication
17	Bo Abrahamsen (32)	Denmark	cohort	female (129029) male (102326)	62.4	thyrotoxicosis	NR	Hip/spine/forearm/humerus	4.3% for thyrotoxicosis/ 1.5% for euthyroid	NR	No Elevated baseline TSH was not associated with an increased risk of hip fracture (HR 0.90; 95% CI, 0.80 to 1.02) or major osteoporotic fractures (HR 0.97; 95% CI, 0.90 to 1.05), nor was subsequent thyroxine prescription predictive of increased risk of fractures.	96% euthyroid/ 4% thyrotoxicosis	Low TSH was significantly more associated with major osteoporotic fractures than normal TSH. patients who present with an elevated TSH, the long-term risk of hip and other osteoporotic fractures is strongly related to the cumulative duration of periods with low TSH—likely from excessive replacement.	Age/chronic comorbid conditions/Fracture history/recent Prednisolone use/Osteoporosis medications use/	**Yes** excessive thyroxine dosing—was significantly associated with an increased risk of both hip fracture (HR 1.09; 95% CI, 1.04 to 1.15) and major osteoporotic fracture (HR 1.10; 95% CI, 1.06 to 1.14)
18	L.J Melton III (33)	USA	Cohort	630 female	42.5 ± 13.25	Thyroidectomy	NR	Vertebra/pelvis/rib/hip forearm	NR	NR	Yes	13.5% hyperthyroid/ 0.47% hypothyroid/ 60.5% euthyroid with adenoma/ 2.69% euthyroid with goiter/ 7.46% with malignancy	There is a little but statistically significant rise in the risk of hip fractures (95% CI 1.01–1.8)	age/ hyperparathyroidism/ osteogenesis imperfecta/ peptic ulcer disease/gastrectomy/malabsorption syndrome/ chronic obstructive lung disease/ renal failure/ rheumatoid arthritis/ hemiplegia/hemiparesis/ parkinsonism/multiple myeloma	NR
19	Margaret C. Garin (34)	USA	cohort	female (2765) Male (2171)	65 years and older	Subclinical hyperthyroidism and hypothyroidism	NR	NR	NR	NR	NR	13.7% hypothyroid/ 84.6% euthyroid/ 1.6% hyperthyroid	There was no association between subclinical hypothyroidism or subclinical hyperthyroidism and hip fracture risk.	Age/BMI/Activity level/Ever-smoker/Alcohol use/Estrogen use/Corticosteroid use/Thiazide use/ no association was found between subclinical hyperthyroidism and incident hip fracture in either sex	NR

### Thyroid cancer

Women with differentiated thyroid cancer (DTC) showed significant changes in Fracture Risk Assessment Tool (FRAX), with a higher increase in the probability of hip fracture than of major osteoporotic fracture (TSH [n.v. 0.3~4.2 mIU/L]: 0.66 ± 1.22 (0.16)) (18). Also, women with a history of hyperthyroidism and thyroid cancer had their first fracture earlier (*P*<0.01) than women without thyroid disease (28), but there were no significant differences between women with thyroid disease and women without thyroid disease in the number or type of fractures (28).

### Hyperthyroidism

Low serum TSH levels (0.1 mU/L) as an indicator of hyperthyroidism in women older than 65 were correlated with higher new hip fractures (20). Males with hyperthyroidism (TSH <0.10 mIU/L) (3, 21) and subclinical hyperthyroidism (17) are at increased risk for hip fracture. Interestingly, thyrotoxicosis, without the aid of other risk factors such as hypogonadism, particularly in men receiving gonadotropin-releasing hormone (GnRH) agonist therapy for prostate cancer, were responsible for the 5-fold increased hip fracture risk in males and 2.1-fold in females (22). Whether treatment of the subclinical syndrome reduces this risk remains unknown (3).

### Euthyroid

In euthyroid older men, TSH and FT4 were not associated with Bone Turnover Markers (BTMs) or hip fracture incidence (27). Lower TSH levels in the euthyroid range were related to lower BMD and weaker femoral structure in elderly women but not men (19). Another study on older men reported that although neither TSH nor FT4 was associated with bone loss, lower serum TSH may be associated with an increased risk of hip fractures (relative hazard [RH] 1.31 per SD decrease in TSH, 95% CI 1.01 – 1.71) (30).

### Thyroid hormone therapy

Women taking thyroid hormone for various thyroid disorders do not appear to have an enhanced prevalence of hip, vertebral, or forearm fractures (28). In another study, excessive thyroxine dosing was significantly but slightly associated with an increased risk of hip fracture (HR= 1.09; 95% CI: 1.04 to 1.15) (32).

### Hypothyroidism

In hypothyroid people, low-energy trauma more likely occurred (71%) compared to 32% of euthyroid subjects (*P*<0.001) (26). Patients with hypothyroidism presenting with fractures are more likely females with low-energy trauma (26). TSH was a predictive factor for fractures in women with subclinical hypothyroidism (23, 24). No statistically significant relation was found between baseline TSH and subsequent fracture risk, but the data suggest a weak positive association with hip fracture risk among women with both low and high TSH (29–32).

### Other outcomes

Lower BMD and FRAX scores for hip and osteoporotic fractures were associated with TPO-Ab in euthyroid postmenopausal women (23). The relative risk of any fractures for patients with thyroidectomy versus their controls was increased 1.5-fold (95% CI, 0.7–3.2) (25). There is a little but statistically significant rise in the risk of hip fractures among thyroidectomized patients (33).

Since some studies focused on women, results may be influenced by involutional osteoporosis (25). Osteoporosis was identified in 90% of hypothyroid subjects who underwent a DEXA scan (26).

### Other risk factors for hip fracture

Risk factors for hip fracture reported to be age (3, 32), sex (3), previous fractures (21, 23, 24, 32), smoking status (3, 17–19, 21, 23, 28–31), alcohol consumption (3, 17, 19, 21, 25, 30), parental fractures (17, 23), body mass index (BMI) (3, 17–19, 21, 23, 28–30), fat mass and weight (17, 20, 23, 25), menopausal status (3, 17–19, 23), disease-free for DTC recurrence, diseases involving bone anti-resorptive therapy (18), vitamin D level (23), calcium/vitamin D supplementation (3, 18), hormone replacement and use of oral estrogen (3, 19, 20), history of hyperthyroidism (3, 20, 22, 25), use of thyroid hormones (3, 20, 25, 32) were among factors related to hip fracture.

Medical history (21, 24, 30, 32), cognitive, visual, and neuromuscular function (21), diabetes mellitus (3, 17, 22, 23), rheumatoid arthritis, hemiplegia, malabsorption syndrome, and gastric surgery, radiotherapy to the pelvis (22), and using medication such as Prednisolone or Osteoporosis medications (3, 22, 24, 30, 32) were among factors correlated with hip fracture. Also, thiazide use, frailty status (3), age at thyroidectomy, extent of surgery (3, 25), menstrual/obstetrical status (28), and physical activity status (3, 29, 30) were related to hip fracture.

## Discussion

We have conducted a systematic literature review to investigate the potential association between thyroid dysfunction and hip fracture outcome. Results indicate that the association of subclinical hypo- and hyperthyroidism with increased risk of hip fracture is still unclear since there is inevitable heterogenicity in the methodology of the studies. Studies were different regarding sample size, follow-up duration, comorbidities, history of previous fracture, history of medication (background therapies), thyroid pathogenesis (thyroid cancer, Goiter, thyroid nodule, autoimmune thyroid disease, etc.), severity of disease, number of events or traumas that occurred, and menopause status in women.

The systematic review and meta-analysis of seven population-based cohorts reported that participants with subclinical hypo- and hyperthyroidism, particularly among those with TSH levels of less than 0.10 mIU/L, compared with euthyroid participants had higher hazard ratios for hip and non-spine fracture but without statistical differences (*P*>0.05) (36). In like manner, all articles mentioned TSH levels of lower than 0.10 mIU/L as a cut off value, however, various articles have reached diffrenet results regarding the association between subclinical thyroid disorders and fractures. A similar meta-analysis study by Zhu et al. investigated 17 prospective cohorts, including 313,557 individuals, and found that subclinical hyperthyroidism contributes to a significantly increased risk of hip, spine, and non-spine fractures by calculating relative risks; however, subclinical hypothyroidism was not associated with risk of any fracture (37). Additionally, in line with our findings, they concluded that age, cutoff value, and follow-up duration might play an important role in BMD, leading to higher fracture risk. Fang et al. evaluated sex-related differences between subclinical thyroid dysfunction and fractures. They demonstrated no significant sex-related differences. Unlike previous studies, they have argued that there is a greater risk of any fracture in men than in women with follow-ups of fewer than ten years; however, the risk of hip fracture was higher in women than men without a significant difference (38).

Mortenson et al., while focusing on the association of different medications with the risk of hip fracture, investigated the impact of thyroid hormone as one of the medications on hip fragility. They reported that patients who were overtreated or undertreated with exogenous thyroid hormone had a significantly higher risk of hip fracture (39). On the contrary, some studies hold up the view that endogenous subclinical hyperthyroidism has more effect on BMD than exogenous (40, 41). Also, Wirth et al. found that excluding all exogenous thyroid hormone recipients and limiting the analysis to individuals with endogenous subclinical hyperthyroidism showed an increased risk from 1.38 to 2.16 for hip fracture (36). A similar work by Ku et al. has demonstrated that TSH suppression therapy after thyroidectomy in postmenopausal women significantly decreased hip, lumbar spine, and femoral neck BMD; conversely, in premenopausal women, significantly increased lumbar spine and femoral neck BMD. Additionally, the case and control groups had no significant difference in men.

Different hypothetical mechanisms have been proposed to illustrate the relationship between thyroid hormone and BMD. First, osteoclasts have receptors for thyroid hormones which can directly influence its function, and since high thyroid hormone results in lower TSH hormone; therefore, besides the direct effect of thyroid hormone, it has an indirect impact on bone turnover and bone loss by regulating TSH (42, 43). Secondly, individuals with subclinical hyperthyroidism seem to have lower thigh muscle strength, possibly leading to increased fall-related fractures (44, 45). Thirdly, unlike osteoclasts, osteoblasts have receptors for both thyroid and estrogen hormones, indicating that these hormones play a crucial role in bone formation. As a result, subclinical hyperthyroidism and low estrogen levels, especially in postmenopausal women, are associated with osteoporosis and an increased risk of fractures (46, 47). Likewise, hypothyrodism has negative impacts on bone health, including reducing bone remodeling, provoking falls, reducing the osteoblast activity and decelerating secondary bone mineralization (5, 48). Notably, there is a possibility that hypothyroid patients who are already on treatment with thyroxine supplements were in fact iatrogenic hyperthyroid (26). Consequently, thyroid hormones profoundly impact BMD (39); however, individuals’ age might have a more important role due to the severity of osteoporosis, the number of traumas or fallings, and the previous history of fractures considerably increasing in elderlies (44). Moreover, many studies do not distinguish between underlying pathogenesis, such as thyroid cancers, thyroid tumors, goiter, thyroid nodules, autoimmune thyroid disease, etc. These conditions affect bone turnover in various ways, possibly responsible for confounding results of included studies and previous reviews.

## Limitation

Different approaches and methodologies were applied in the included studies, resulting in significant heterogenicity. For instance, different follow-up duration, a wide variety of statistical analysis reports (hazard ratio, relative risk ratio, odds ratio, etc.), and the absence of clear control cases limited our interpretation. Additionally, there is an increase in the upper physiological TSH reference range with age (e.g. 97.5 percentile from 4.32 mUI/l at the age of 20-30 to 5.23 mUI/l around the age 80 and 5.71 mUI/l around age of 90). Thus, some older individuals (i.e. with an increased risk of fracture) may be misclassified as having subclinical hypothyroidism, while their TSH may be indeed within their age-specific reference range. Plus, considering the conditions in which the thyroid hormones are evaluated is very important. For instance, assessing hormone levels right after the fracture is not recommended since fractures can be one of the triggers of acute stress and a contributing factor to the change in TSH levels. Furthermore, selection bias may be present despite our efforts not to set a strict and narrow inclusion criterion. Nevertheless, it is essential to study the available literature to reach a consistent conclusion and recognize the gaps that still need to be addressed.

The main strength of this study is that, in contrast to recent studies to find a positive trend for the impacts of subclinical thyroid dysfunction on hip fracture, our study tried to avoid biases and report reliable evidence in this matter. In this regard, we did not exclude studies due to heterogeneity or contradicted results. For future studies, we recommend that studies share their data in valid and authorized data banks to help big data scientists perform more detailed stratified analysis.

## Conclusion

Reaching a consensus conclusion is not feasible regarding the association between subclinical thyroid dysfunction and hip fracture due to the heterogenicity of evidence, but we believe that confirming thyroid dsyfunction as a validated risk factor for hip fracture is yet to come. More studies with clear control selection are required to shed light on this matter which adjusts all possible potential confounders such as sex, age, endogenous or exogenous thyroid hormone, follow-up duration, age-adjusted cutoff values, body weight, cigarette smoking, previous fracture, and the epidemic of falls.

## Data availability statement

The raw data supporting the conclusions of this article will be made available by the authors, without undue reservation.

## Author contributions

(1) The conception and design of the study: EM, SS (2) Acquisition of data: SY, MD, AG (3) Analysis and interpretation of data: HS, AM (4) Drafting the article: EM, SM, KQ, GA, SP, MA, PM (5) Revising it critically for important intellectual content: SS, SY, OD (6) Final approval of the version to be submitted: SS, EM, OD. All authors contributed to the article and approved the submitted version.
